# Relationship between Multimorbidity and Quality of Life in a Primary Care Setting: The Mediating Role of Dyspnea

**DOI:** 10.3390/jcm11030656

**Published:** 2022-01-27

**Authors:** Pietro Alfano, Giuseppina Cuttitta, Palma Audino, Giovanni Fazio, Sabina La Grutta, Salvatore Marcantonio, Snamid Palermo Cooperative Group, Salvatore Bucchieri

**Affiliations:** 1 Institute for Biomedical Research and Innovation (IRIB), National Research Council of Italy, 90146 Palermo, Italy; giuseppina.cuttitta@irib.cnr.it (G.C.); palma.audino@hotmail.it (P.A.); salvatore.bucchieri@irib.cnr.it (S.B.); 2 Institute of Traslational Pharmacology (IFT), National Research Council of Italy, Via Fosso del Cavaliere 100, 00133 Roma, Italy; 3 Triolo Zanca Clinic, Piazza Fonderia 23, 90133 Palermo, Italy; faziogiova@gmail.com; 4 Department of Psychology, Educational Science and Human Movement, University of Palermo, 90128 Palermo, Italy; sabina.lagrutta@unipa.it; 5 Quality, Planning and Strategic Support Area, University of Palermo, Piazza Marina 61, 90133 Palermo, Italy; salvatore.marcantonio@unipa.it; 6 Snamid Palermo Cooperative Group Papa Pio X 18/20, 90142 Palermo, Italy; presidenza.palermo@snamid.it

**Keywords:** dyspnea, multimorbidity, primary care, quality of life, airway obstruction

## Abstract

Multimorbidity is known to impair Quality of Life (QoL) in patients in a primary setting. Poor QoL is associated with higher dyspnea perception. How multimorbidity and dyspnea perception are related to QoL needs clarification. The aim of the present study is to evaluate the mediating role of dyspnea perception in the relationship between multimorbidity and QoL in adults with and without airflow obstruction in a primary care setting. Seventeen general practitioners participated in the study: a total of 912 adult patients attending the practitioner’s surgery for a generic consultation completed a preliminary respiratory screening; 566 of them answered a respiratory questionnaire between January and June 2014, and 259 of the latter (148 M, aged 40–88) agreed to go through all the of procedures including spirometry, the IMCA and QoL (SF-36 through Physical Health “PCS” and Mental Health components) questionnaires, evaluation of comorbidities and the mMRC Dyspnea Scale. For screening purpose, a cut-off of FEV_1_/FVC < 70% was considered a marker of airflow obstruction (AO). Of the sample, 25% showed airflow obstruction (AO). No significant difference in mMRC score regarding the number of comorbidities and the PCS was found between subjects with and without AO. Multimorbidity and PCS were inversely related in subjects with (*p* < 0.001) and without AO (*p* < 0.001); mMRC and PCS were inversely related in subjects with (*p* = 0.001) and without AO (*p* < 0.001). A mediation analysis showed that the relation between number of comorbidities and PCS was totally mediated by mMRC in subjects with AO and partially in subjects without AO. We conclude that the effect of multimorbidity on PCS is totally mediated by mMRC only in AO. Detecting and monitoring mMRC in a primary care setting may be a useful indicator for evaluating a patient’s global health.

## 1. Introduction

The number of people with chronic diseases and multiple comorbidity is growing with increasing life expectancy [1,2]. As a consequence, chronic airway diseases characterized by airflow obstruction (AO), such as asthma or chronic obstructive pulmonary disease (COPD), contribute significantly to worldwide morbidity [3,4] with a significant impact on health status in the general population. In an epidemiological study, among the Italian general population, about 18% of subjects presented airway obstruction, as assessed by spirometry [5]. Patients with airflow limitation often reported respiratory symptoms such as chronic cough, sputum production, wheezing, breathlessness and exercise limitation [4].

Primary health care and general practitioners have a crucial role in the diagnosis and management of chronic airway disease [6]. In this context, dyspnea was one of the main symptoms, only second to pain; it is estimated that up to a quarter of the general population is affected by it [7,8,9]. Dyspnea is a disabling symptom in patients with respiratory diseases and in particular in patients with airway chronic obstruction. It was defined as ‘‘subjective experience of breathing discomfort that consists of qualitatively distinct sensations that vary in intensity’’ [10]. In adults with airflow obstruction, such as COPD patients, dyspnea is reported as the most important determinant of a low physical and mental health component [11,12], influencing daily life functions [13]. Moreover, dyspnea perception becomes worse over time in COPD patients and is related both to progressive airflow limitation and to the worsening of diffusing capacity (DLCO) or psychological state [14]. Poor QoL is associated with a higher level of dyspnea perception. Additionally, patients with comorbidities had a poor self-reported health status [15] and a poorer quality of life [16].

Depression status, airway inflammation, older age and severity of asthma influence dyspnea perception [17]. Moreover, in asthma patients, dyspnea is related to anxiety. [18] and chronic panic disorder [19]. The presence of comorbidities is more common in patients with chronic obstructive airway diseases when compared with the general population [20] and affects QoL [21]. In particular, in patients with multiple comorbidities, even when Asthma and COPD are considered separately, a worsening in QoL and affective well-being has been noted [21,22,23]. Patients with COPD are at high risk for functional limitations, unhealthy behavior, poor mental status and poor QoL [24].

Multimorbidity, which is defined as the coexistence of two or more chronic health conditions in an individual and in which the frequency increases with ageing, is indeed a critical challenge in health care settings, including primary care [25]. Fortin et al. showed that physical health worsens more than mental health with increasing morbidities in primary-care adult patients, even when confounding variables were taken into consideration [26]. Furthermore, a metanalysis reported that QoL deteriorated with an increasing number of chronic diseases. While QoL is one of the most important outcomes in patients with multimorbidity, nevertheless, the exact relationship between QoL and comorbidities is still unclear [27] and further longitudinal studies could be useful to clarify this complex relationship [28].

Ageing plays an important role in impairing QoL. A poor health-related quality of life or HRQoL has been associated with reduced activities of daily living, a higher frequency of hospitalization and increased mortality [29]. A negative effect of age on physical function, role limitations and general health was reported by Haraguchi et al. [30]. Similarly, an inverse effect of age on QoL was found in patients with asthma [31]. Conversely, Martinez et al. [32] showed that patients with COPD who were older reported better QoL than younger ones, probably due to the effect of tolerance and adaptation to the disease.

How comorbidities and dyspnea influence the health-related quality of life (QOL) in patients with airway obstruction need further studies. The treatment of dyspnea has a key role in the management of respiratory patients in a primary care setting. In a recent review, dyspnea is defined as a complex, subjective sensation related to comorbidities, hormonal changes and mood swings as well as the underlying respiratory cause [33].

We hypothesize that dyspnea perception mediates the associations between number of comorbidities and quality of life, both in its physical (PCS) and mental components (MCS), in patients with airway obstruction. The present study aims to determine the mediating role that dyspnea perception, evaluated by mMRC, has in the relationship between the number of comorbidities and QoL in adults with and without obstruction in a primary care setting.

## 2. Materials and Methods

### 2.1. Study Design

**Figure 1:**
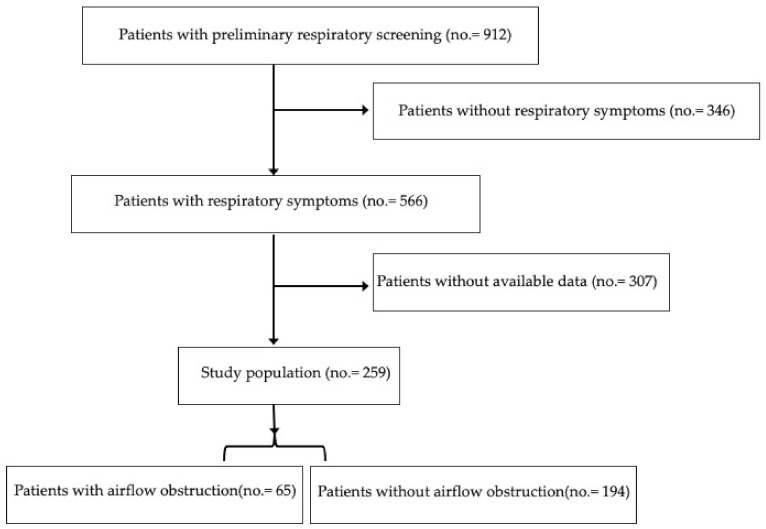
Flow of the present study.

Seventeen general practitioners (GPs) participated in the study during January–June 2014; 912 patients, who went to surgeries for a generic consultation, completed a preliminary respiratory screening; 566 reported respiratory symptoms; and of these, 259 went through all the procedures (Figure 1). None of the subjects were nursing home residents. The procedures of the study included the following: (1) indicators for monitoring COPD and asthma in the EU questionnaire (IMCA questionnaire) [34]; (2) comorbidity and hospitalization evaluations; (3) dyspnea perception on the modified Medical Research Council dyspnea scale (mMRC) [35]; (4) Quality of Life questionnaire (SF-36) [36]; and (5) spirometry. The study was approved by the Local Institutional Ethics Committee (No. 10/2013, 18 September 2013). All subjects gave written informed consent. The individual privacy of clinical data was guaranteed under Italian law.

### 2.2. Procedures

Respiratory Health Questionnaire: Preliminary respiratory evaluation was investigated using the Indicators for monitoring COPD and asthma in the EU questionnaire (IMCA questionnaire) [34].

The Modified British Medical Research Council questionnaire (mMRC) was also used. Breathlessness is a complex subjective sensation that is an important feature of respiratory disease. Dyspnea perception was evaluated by mMRC, a short questionnaire that allows for a numeric value to be placed on each subject’s exercise capacity. It uses a scale from 0 to 4: 0, no breathlessness except on strenuous exercise; 1, shortness of breath when hurrying on the level or walking up a slight hill; 2, walks slower than people of the same age on the level because of breathlessness or has to stop to catch breath when walking at their own pace on the level; 3, stops for breath after walking ~100 m or after a few minutes on the level; and 4, too breathless to leave the house, or breathless when dressing or undressing. It was administered by an interviewer with the statements framed as questions. The mMRC questionnaire is an instrument easily understood by patients and is quick to fill out.

### 2.3. Smoking History

Smoking status included never smokers (<100 cigarettes), former smokers (quit > 12 months before data collection) and current smokers (quit < 12 months before diagnosis or currently still smoking). Smoking pack years defined as packs/y (1 pack = 20 cigarettes) years of smoking were directly garnered during the assessment [5].

Allergy was defined as positive skin prick test for common inhalant allergens in Mediterranean area.

### 2.4. Spirometry

Height and weight were measured in all patients in standing position without shoes, using a stadiometer and an electronic digital scale: BMI was computed as weight/height^2^ (kg/m^2^). Pulmonary function tests were performed using a portable spirometer (MicroLoop, Micro Medical, Chatham Maritime, Kent, UK). Forced expiratory volume in one second (FEV_1_), forced vital capacity (FVC) and maximum mid-expiratory flow (FEF_25–75%_) were measured according to ATS/ERS guidelines [37], and the best FVC and FEV_1_ were retained. Spirometric predicted values were those from the Global Lung Initiative. Airflow obstruction was defined as FEV_1_/FVC lower than 70% [38,39].

The SF-36 questionnaire. QoL was assessed using a 36-item Short Form Survey (SF-36) through the two component factors Physical Health (PCS) and Mental Health (MCS). The questionnaire, consisting of 36 items, was summarized and transformed to give eight summary scales measuring health concepts: physical functioning (PF), role-physical limitation (RP), bodily pain (BP), general health perception (GH), vitality (VT), social functioning (SF), role limitation attributable both to emotional problems (RE) and mental health (MH). The PF, RP and BP scales were considered the primary components of the physical component summary (PCS) score, whereas the SF, RE and MH were considered the primary components of the mental component summary (MCS) score. The GH and VT were considered components of both dimensions.

### 2.5. Hospitalization and Comorbidities

Hospitalization and comorbidities were evaluated from GPs’ files. A patient card containing the pathologies and history of hospitalizations that he/she was suffering from was only provided by GPs to the interviewer for each patient that provided consent for all of the procedures. The chronic medical conditions for all patients enrolled in the study were compiled and counted, including those grouped into cardiovascular or respiratory diseases. Multimorbidity was measured with a simple count of the number of chronic diseases.

### 2.6. Data Analysis

All statistical analyses were performed using R-open source software package (R Core Team—Version 4.1.2).

Prevalence and measures of central tendency were used to describe the anthropometric and clinical data. The SF-36 questionnaire raw scores were converted into standard scores (Mean ± SD = 50 ± 10) through the Access 2007 SF-36 program in accordance with the questionnaire’s guidelines [36]. Hospitalization was evaluated by the presence or absence of an event. Comorbidity numbers were evaluated by the sum of all the comorbidities of each patient. Chronic medical conditions for all patients enrolled in the study were compiled and counted, including those grouped into cardiovascular or respiratory diseases. Multimorbidity was defined by two chronic physical conditions, in line with previously used definitions [40]. The number of comorbidities was treated as a dichotomous variable using the median value <3> in order to obtain more accurate estimates. The mMRC score was considered as an ordinal variable from 0 to 4. We categorized mMRC using the value 2, whereas none of the participants indicated 4; a rating of 3 was reported by a small number of participants, so levels 3 and 2 were merged. The correlation analysis was performed to analyze the relationships between the study variables. The mediation hypotheses were tested according to Baron and Kenny’s approach [41], through a mediation analysis in patients with airflow obstruction and patients without airflow obstruction separately. The total, direct and indirect effects of comorbidity numbers on PCS through mMRC were evaluated by mediation using the R Package for Causal Mediation Analysis [42]. To evaluate the significance of the mediation model, we used bootstrapping [43]. This procedure does not make the assumption of normality on the sampling distribution of indirect effects and retains high power while maintaining adequate control over the Type I error rate [44,45,46]. The test is statistically significant (at 0.05) if both confidence limits have the same sign. This indicates that the null hypothesis of a null indirect effect has to be rejected. A bootstrapping procedure was used (with 5000 bootstrap samples) to estimate the 95% confidence interval for the indirect (mediated) effect [43].

## 3. Results

General patient characteristics: 566 patients reported respiratory symptoms on the basis of answers to a preliminary respiratory screening; 259 of these (46%) agreed to go through all the procedures, with 148 males (57%) and 111 females (43%) aged 40–88 years (mean 65.65 SD 10.15, median 67). Anthropometric and clinical characteristics are shown in Table 1 and Table 2.

**Figure 2:**
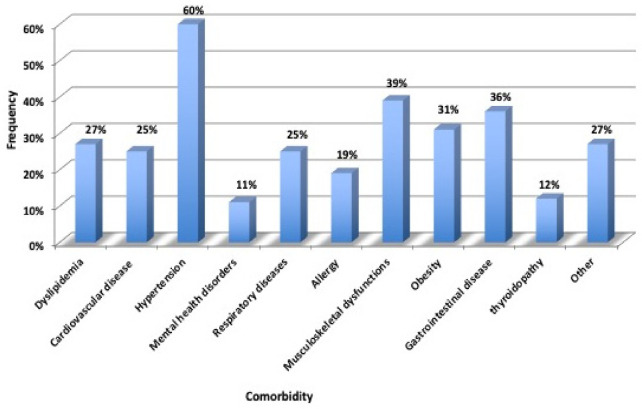
Comorbidity frequency in the overall sample.

**Figure 3:**
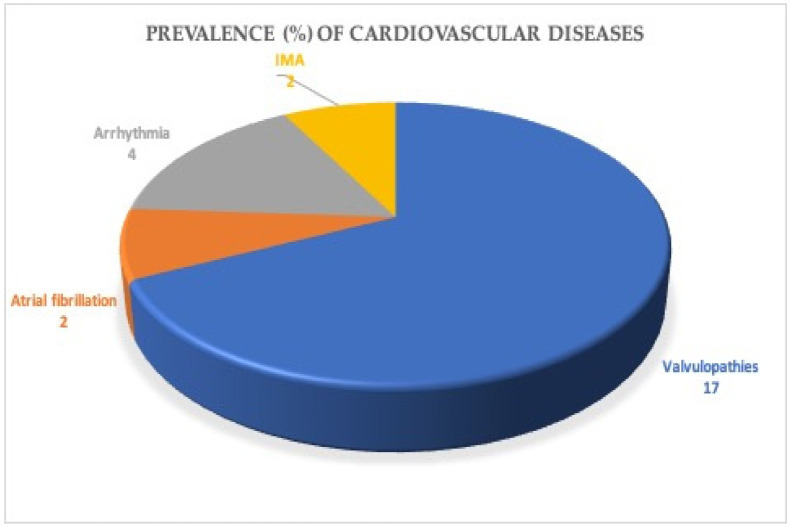
Prevalence (%) of cardiovascular diseases in the overall sample.

Comorbidities are reported in Figure 2. The most common chronic comorbidities were dyslipidemia (i.e., hypertriglyceridemia and hypercholesterolemia), cardiovascular diseases, hypertension, mental health disorders (i.e., depression and anxiety), respiratory diseases (i.e., COPD, asthma, obstructive sleep apnea syndrome (OSAS) and bronchiectasis), allergy (as a positive skin prick test for inhalant allergens), musculoskeletal dysfunctions (i.e., osteoporosis, arthropathy and low back pain), obesity, gastrointestinal disease (i.e., gastritis, colitis and hiatal hernia), thyroidopathy and others. The prevalence of cardiovascular disease included valvulopathies; atrial fibrillation; arrhythmia as the presence of bradycardia, tachycardia or extrasystoles; and history of acute myocardial infarction (IMA) and is reported in Figure 3. The number of comorbidities was evaluated as the sum of all the comorbidities of each patient. Hypertension, musculoskeletal dysfunctions and gastrointestinal disease were the most commonly reported diseases. Almost all subjects (97%) had one or more comorbidities, and more than half of the patients had at least four comorbidities. Of the 259 patients, 148 (57%) reported a history of hospitalization. For the mMRC scale, none of the participants indicated a score of 4, a score of 3 was reported for 2%, 16% indicated a score of 2, 55% indicated a score of 1 and 27% indicated a score of 0.

In the overall sample, 65 subjects (25%) showed airflow obstruction while 194 (75%) were without airflow obstruction. As concerns QoL, a Mann–Whitney U test showed a significant difference in PCS between males and females (*p* = 0.000): females had a lower PCS than males. The same result was found for MCS with a sample *t*-test (*t*(257) = −3.505; *p* = 0.001), in which females had a lower MCS than males, as in the general population. No significant differences were found between obstructed and unobstructed subjects concerning PCS (Mann–Whitney U = *NS*) and MCS (Sample *t*-test = *NS*) for either mMRC or number of hospitalizations and comorbidities (Mann–Whitney U = *NS*). No significant PCS differences were found between smokers (current or ex) and nonsmokers (Mann–Whitney U = *NS*). By contrast, MCS (*t*(255) = 2.34; *p* = 0.020) was significantly different in two groups: nonsmokers had an MCS higher than smokers. No significant differences were found between smokers (current or ex) and nonsmokers concerning mMRC (Mann–Whitney U = *NS*) and between number of hospitalizations and comorbidities (Mann–Whitney U = *NS*).

### Model of Mediating Effects

In accordance with our hypothesis, we investigated the relationship between study variables by evaluating the mediating role of mMRC. We ran a mediation model to derive the total, direct and indirect effects of number of comorbidities on QoL, checking for gender, age and smoking habits in both groups. The PCS and MCS index was used to assess QoL; the mediation model was used separately to investigate the mediating effect on both the physical and mental components of QoL. Nevertheless, assumptions were not respected when we tested MCS as a dependent variable so the mediation hypothesis was not confirmed for the mental component of QoL (results not shown). In accordance with our hypothesis, we used two separate models with PCS as a dependent variable: one for AO subjects and the other for non-AO subjects.

**Figure 4:**
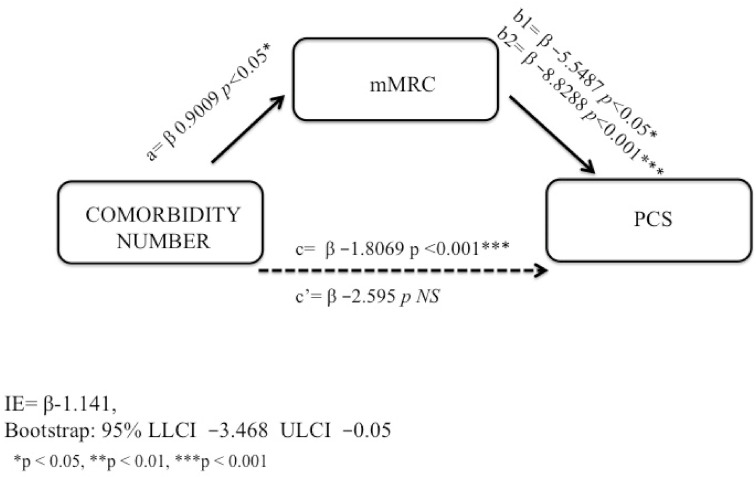
Mediating role of dyspnea in adults with airway obstruction.

Correlations among the study variables in AO and non-AO subjects were examined (Table 3 and Table 4). Adults with airflow obstruction: the first model was used on AO subjects. In the final regression model for PCS as a dependent variable in obstructed subjects, only an indirect effect through dyspnea perception emerged as a significant and inverse predictor of PCS but not a direct effect of number of comorbidities (Table 5). The total effect is composed by direct and indirect effects. The bootstrapping results for the indirect effect were significant as indicated by the 95% not crossing over “0” (Figure 4). The model was checked for confounding variables: age, gender and smoking habits, which were not significantly related to PCS (NS, respectively). Therefore, in AO subjects, we found that the mMRC was a total mediator in the association between number of comorbidities and PCS because the number of comorbidities was no longer related to PCS after mMRC was inserted in the model as a mediator (Figure 4).

**Figure 5:**
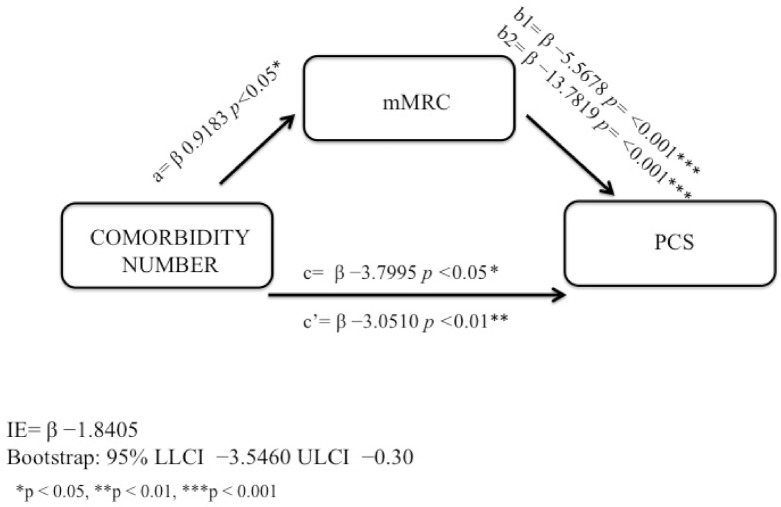
Mediating role of dyspnea in adults without airway obstruction.

Adults without airflow obstruction: The same analysis was performed in subjects without AO; dyspnea was evaluated as the mediating factor between number of comorbidities and PCS. Correlations among the study variables in subjects without AO were examined (Table 3). In the final regression model for PCS as a dependent variable in unobstructed subjects, both a direct effect of the number of comorbidities and an indirect effect through dyspnea perception emerged as significant inverse predictors of PCS (Table 6). The total effect is composed by direct and indirect effects; bootstrapping results for the indirect effect were significant as indicated by the 95% not crossing over “0” (Figure 3). The model was checked for confounding variables: age gender and smoking habits, which were not significantly related to PCS (NS). Therefore, a partial mediating role of mMRC was found in subjects without AO because the number of comorbidities was still a significant predictor of PCS after mMRC was inserted in the model as a mediator (Figure 5).

## 4. Discussion

In this real-life observational study, a mediation analysis was used to clarify the relationship between dyspnea assessed with mMRC scale, multimorbidity and HRQoL through the two factor structures of physical and mental components, which are based on a summary of the SF-36 in primary care. PCS and MCS were confirmed as a valid instrument for the assessment of quality of life related to physical and mental health, respectively [47], whereas SF-36 has generally been used in many studies [48,49] as a useful tool for clinical management. To overcome the conceptual limitation of classically defined comorbidity, the concept of multimorbidity has been proposed. It refers to the coexistence of multiple conditions without distinction between a “principal” disease and associated diseases. In accordance with our hypothesis, we used the mediation model in adults with and without airway obstruction, respectively. We found a strong mediating effect of dyspnea on the relationship between multimorbidity and HRQoL only in adults with airway obstruction. When dyspnea was included as a mediator, the relationship between number of comorbidities and HRQoL became insignificant in adults with airways obstruction. Conversely, when dyspnea was included as a mediator in adults without airflow obstruction, the relation between number of comorbidities and HRQoL remained significant, reflecting the fact that it was a partial mediating factor in the relationship between multimorbidity and HRQoL. The novel finding of our study is the indirect mediating effect of dyspnea in the association between number of comorbities and HRQoL. We used this mediation model because of the complex relationship between HRQoL, on the one hand, and because of the comorbidities and dyspnea perception, on the other. For this reason, the mediating variable helps to explain the process through which two variables are related [41]. With reference to the theoretical model [41] on which this is based, the mediation analysis further increased our understanding of this relationship when compared with the regression analysis results. These findings could help clarify the possible underlying mechanisms about the complex role of dyspnea and its different impacts in subjects with and without airway obstruction. These results may also suggest that dyspnea grades give a good estimate of HRQOL in patients with multimorbidity.

The higher the number of chronic diseases and degree of dyspnea, the poorer the reported physical health. Multimorbidity indirectly influences physical health through dyspnea. This result indicates that improving patients’ physical health has great potential in helping them achieve a good quality of life. Moreover, dyspnea is one of the major symptoms that impacts the quality of daily life [50] and reflects the patient’s perspective of their disease, and this is a key outcome in the disease management, especially in the presence of airway obstruction. However, we did not find a mediating effect of dyspnea perception on MCS, probably due to the fact that patients tend to hide psychological problems that could harm their social acceptability. This could lead to an underestimation of the mental scores of the SF-36. Moreover, Damarell et al. [51] revealed that, when it is measured by a simple count of chronic conditions, multimorbidity was adversely related only to physical functioning, thus limiting the ability to find causal relationships.

In our study, the average SF-36 scores were only 46.9 for PCS and 45.7 for MCS, which meant that the HRQoL of our patients was not high. Patients with multiple chronic diseases experienced a high level of physical and functional distress, which has a strong impact on their health status [52]. Our finding about the role of dyspnea was consistent with those of a recent review [52] proving the association between decreased functional status and worse psychological health in older individuals. Previous studies highlighted factors that can lead to dyspnea and impaired health-related quality of life in COPD patients [53,54,55]. The presence of dyspnea, reduced physical activity, concomitant chronic conditions, reduced mobility, and a high level of anxiety could be involved. In particular, in COPD patients, there has been a decreased quality of life related to a reduced physical activity reported [56,57]. People who are breathless often avoid physical activities [58], many becoming socially isolated. This also supports our findings regarding the relevancy of physical dimension of HRQoL. In addition, the experience of breathlessness was linked to intolerance to physical activity and was distressing and debilitating for patients, affecting their quality of life [59]. A recent study reported that respiratory symptoms are among the commonest health problems, leading to the risk of developing disease, in the general population [60]. Moreover, breathlessness was associated with impairment in PCS and MCS, in particular in patients without asthma or COPD [60]. In asthmatics, a high level of anxiety was found to be responsible for the escalation in dyspnea during the progression of the disease, whereas low anxiety may protect them against increases in dyspnea [61]. In agreement with a recent study [2], we found an average of 3.7 co-existing chronic conditions per patient. Cardiovascular, obesity, gastrointestinal and musculoskeletal diseases were amongst the commonest conditions. Unfortunately, comorbidities are often underrecognized and undertreated: this explains the differences in their prevalence among studies [62,63]. Comorbidities are frequent in chronic obstructive pulmonary disease and significantly impact patients’ quality of life, exacerbation frequency and survival. Moreover, there is increasing evidence that certain diseases occur in greater frequency amongst patients with chronic obstructive pulmonary disease than in the general population and that these comorbidities significantly impact patients’ health outcomes [64,65].

Multimorbidity was associated with poor self-reported health status, greater health service needs, poorer quality of life and higher mortality rates [16]. In our study, multimorbidity was confirmed to be related to lower HRQoL, highlighting the importance of investigating dyspnea perception. This promotes the idea of focusing more on health status as shortness of breath and severity of disease; these can be linked to reduced physical activity, loss of muscle mass and strength and problems with mobility, leading to a condition of frailty [66]. In addition, fear of dyspnea and fear of physical activity were related to decreased gray matter, which might negatively influence the course of disease and may impact quality of life. A Cochrane review focused on improving the outcomes in patients with multimorbidity in primary care showed that interventions are more successful if they are linked with functional outcomes rather than disease specific outcomes [67]. An increasing number of chronic diseases was related to a poorer quality of life, revealing [40,68] a ceiling effect between two and three chronic conditions, while another study found that four or more conditions had a significant effect on quality of life compared with less than three chronic conditions [69]. Our results suggest that multimorbidity requires particular attention due to the association between the number of chronic conditions and dyspnea rate. The use of a dyspnea perception questionnaire should lead us to pay more attention to breathlessness perception and help patient management. According to our results, dyspnea may be present at any age; nevertheless, strategies to improve HRQL should consider the different impacts of different factors across age groups [32]. Our findings support a clinical use of mMRC in a primary care setting as a tool for evaluating patients’ overall health and in particular their physical health status [70].

## 5. Limitations

The study had some limitations that must be considered in the interpretation of our findings. The leading limitation was linked to the definition of airflow obstruction. In the literature, the main criterions used were the FEV_1_/FVC < 0.70 proposed by the Global Initiative for Chronic Obstructive Lung Disease (ATS-GOLD) committee [70]; the lower limit of normal (LLN) according to the ERS definition with predicted values was calculated using regression equations [38,71]. The activities of the GOLD guidelines have supported using fixed ratios as a consensus definition for airflow obstruction. However, some may argue that this simple ratio tends to over-diagnose the disease in older adults, as the fixed ratio is associated with increased risk of death whereas using the lower normal limit is not [72]. However, the wide confidence interval of LLN increased the likelihood of underdiagnosis of COPD in older individuals and in symptomatic smokers [73]. Previous studies investigated subjects in between the two definitions of airway obstruction and showed that their clinical profile is characterized by marked comorbidity and a poor health-related quality of life [74,75]. The literature contains a similar number of articles endorsing each of these competing definitions [76]. Moreover, the main purpose of the present study was to define the relationship of multimorbidity and dyspnea in subject with and without airflow obstruction. A recent metanalysis showed that individuals with airflow limitations, when evaluated with LLN or fixed-ratio criteria, show no significant difference regarding the risk of developing comorbidities [77]. According to Celli, the use of the fixed threshold of FEV1/FVC < 0.70 can be considered a useful, simple tool that has prognostic and practical value, thus helping to remove barriers to widespread use of spirometry [73,78]. In the present study, the sample was limited to patients living in an urban area of Sicily, which is a limitation when analyzing the results is the geopolitical context; therefore, a generalization of the findings to other contexts should be taken with caution. Another important aspect concerns the absence of socio-economic variables associated with our patients. Failure to adjust for these variables may have introduced a confounding bias. Some variables assessed in our study were based on self-reporting and thus a reporting bias (recall and social desirability) is possible. Indeed, the SF-36 is a generic quality-of-life instrument rather than a respiratory-specific questionnaire. Using administrative data for comorbidities could have led to some medical conditions being under-represented, but any selection of diseases would inevitably be arbitrary. In our analysis, we used the number of chronic conditions; future studies should examine more objective measures of multimorbidity as well clarify the progression and severity of conditions and examine whether a greater contribution to the results is due to some conditions rather than others. Causal interpretations of associations necessitate within-subject comparison over time. Dyspnea is already an important cause of morbidity in both prevalence and impact [10,79,80]. Additionally, dyspnea is a multifactorial symptom, involving the interaction between various physiological, psychological and environmental factors. Moreover, mMRC was assessed by self-report of respondents, while the literature suggests that people are likely to rate their dyspnea as less severe than clinicians using the mMRC [81,82,83]. Nevertheless, future studies should use more elaborate instruments to assess multidisciplinary and multidimensional aspects of dyspnea as well. Mediation analyses do not allow for causal inferences yet suggest a possible pathway of associations that should be investigated in future longitudinal or experimental studies. Finally, by focusing on different forms of airflow obstruction, we may have failed to recognize specific patterns of respiratory diseases.

## 6. Conclusions

We conclude that the effect of the number comorbidity on PCS is completely mediated by mMRC only in subjects with airflow obstruction. Detecting and monitoring mMRC in a primary care setting seems to be a useful indicator for evaluating a patient’s overall health. Indeed, to evaluate a comprehensive disease-specific health status, alongside spirometry measurement, patient-reported indications of disease severity, mMRC for example should also be considered. Multimorbidity, measured by the number of multiple diseases in a primary care setting, is associated with quality of life after adjusting for age, gender and smoking habits. The direct and indirect mediating effect of dyspnea in the association between number of comorbities and QoL was found in subjects with and without airflow obstruction. Accurate assessment and diagnosis, and greater awareness and understanding of related factors can facilitate targeted treatments of dyspnea and subsequently dramatically improve clinical outcome.

This study suggests that detecting risk factors and pathways common to multimorbidity and functional impairment could aid in designing active disease management, focusing interventions to delay, and preventing or alleviating age-related health deterioration. Moreover, the evaluation of MRC dyspnea scales in clinical practice is strongly recommended to manage subjects with pharmacological, rehabilitative and educational strategies and to relieve their symptoms. Our results suggest that multimorbidity patients require particular attention due to the association between the number of chronic conditions and a complex condition such as dyspnea, in which an evaluation could help clinicians to determine the needs of subjects and to thus optimize their treatment. 

## Figures and Tables

**Table 1:** Anthropometric and clinical characteristics of the sample.

	N (Valid %) or Mean (SD) or Median
Male	148 (57%)
Age	65.65 (10.15)
Body mass index (kg/m^2^)	28.3 (4.8)
Current smokers	81 (31%)
Former smokers	119 (46%)
Airflow obstruction	65 (25%)
Comorbidity number	3 Mdn
Patients with history of Hospitalization (>1)	148 (57%)
SF36 PCS	46.9 (9.1)
SF36 MCS	45.7 (9.5)

**Table 2:** Prevalence for mMRC levels.

	No. (Valid %)
0	70 (27%)
1	143 (55%)
2	42 (16%)
3	4 (2%)
4	0 (0)

**Table 3:** Spearman correlation between study variables in AO subjects.

Variables	1	2	3	4
1. Comorbidities (no.)	1			
2. mMRC	0.399, *p* = 0.001 **	1		
3. SF36 (PCS)	−0.296, *p* = 0.017 *	−0.441, *p* < 0.001 **	1	
4. SF36 (MCS)	−0.139 *NS*	−0.259 *p <* 0.05 *	−0.039 *NS*	1

* *p* < 0.05. ** *p* < 0.01.

**Table 4:** Spearman correlation between study variables in non-AO subjects.

Variables	1	2	3	4
1. Comorbidities (no.)	1			
2. mMRC	0.246, *p* = 0.001 **	1		
3. SF36 (PCS)	−0.323, *p* = 0.000 ***	−0.517, *p* = 0.000 ***	1	
4. SF36 (MCS)	0.082 *NS*	−0.076 *NS*	−0.114 *NS*	1

** *p* < 0.01. *** *p* < 0.001.

**Table 5:** Total direct and indirect effect—AO group.

Independent Variable	Dependent Variable	β	95% CI Lower	95% CI Upper	*p*
Total effect					
comorbidity number	SF36 (PCS)	−3.737	−7.489	−0.17	0.036 *
Direct effect					
comorbidity number	SF36 (PCS)	−2.595	−5.817	0.69	0.144
Indirect effect					
comorbidity number	mMRC	−1.141	−3.468	−0.05	0.049 *

* *p* < 0.05.

**Table 6:** Total direct and indirect effect—non-AO group.

Independent Variable	Dependent Variable	β	95% CI Lower	95% CI Upper	*p*
Total effect					
comorbidity number	SF36 (PCS)	−4.8915	−7.6929	−2.27	0.000 ***
Direct effect					
comorbidity number	SF36 (PCS)	−3.0510	−5.2455	−0.79	0.001 **
Indirect effect					
comorbidity number	mMRC	−1.8405	−3.5460	−0.30	0.02 *

* *p* < 0.05. ** *p* < 0.01. *** *p* < 0.001.

## Data Availability

The primary data are available upon request.

## Supplementary Materials

The following supporting information can be downloaded at: https://www.mdpi.com/article/10.3390/jcm11030656/s1.
