# Supplementary material for: Relationship between Multimorbidity and Quality of Life in a Primary Care Setting: The Mediating Role of Dyspnea
Source: J Clin Med. 2022 Jan 27;11(3):656. doi: 10.3390/jcm11030656 (PMC8837036; doi:10.3390/jcm11030656)
Supplement: Supplementary file 1 [file jcm-11-00656-s001.zip › jcm-1542717-supplementary.pdf]

Snamid Palermo Cooperative Group:

Orlando Luigi; Petrona Baviera Francesco; Accardo Palumbo Vincenzo; Messina Giovanni; Perrone Rosario; Accardo Maria; Barcellona Vincenzo; D'Ippolito Giuseppe; Di Bella Antonina; Di Maso Natale; Genovese Giovanni; Giambruno Ornella; Sinaguglia Giuseppina; Sofia Giacomo; Sparacino Giosué; Tarantino Natale; Montalto Antonino
